# The complete chloroplast genome sequence of *Begonia ferox*, an endangered species in China

**DOI:** 10.1080/23802359.2023.2231103

**Published:** 2023-07-18

**Authors:** Yangming Zhou, Xuan Yang, Qiongyue Liang, Tao Deng, Xinghua Hu

**Affiliations:** a College of Tourism and Landscape Architecture, Guilin University of Technology, Guilin, China; b Guangxi Institute of Botany, Guangxi Zhuang Autonomous Region and Chinese Academy of Sciences, Guilin, China

**Keywords:** *Begonia ferox*, Begoniaceae, chloroplast genome, phylogenetic relationship

## Abstract

*Begonia ferox* C.I Peng & Yan Liu (2013) was rated as endangered according to Red List of Chinese Plants. In this study, we report the complete chloroplast genome of *B. ferox*. The chloroplast genome is 169,114 bp in length as the circular, with the GC content of 35.5%, composed by a large single-copy (LSC) region of 75,887 bp, a small single-copy (SSC) region of 18,105 bp, and two inverted repeat regions (IRs) of 37,561 bp in each. The genome comprises 174 encoded genes in total, including 114 protein-coding genes, eight ribosomal RNA genes, and 52 transfer RNA genes. Phylogenetic analysis indicated that *B. ferox* is genetically closest to *B. gulongshanensis*.

## Introduction

**Figure 1.**
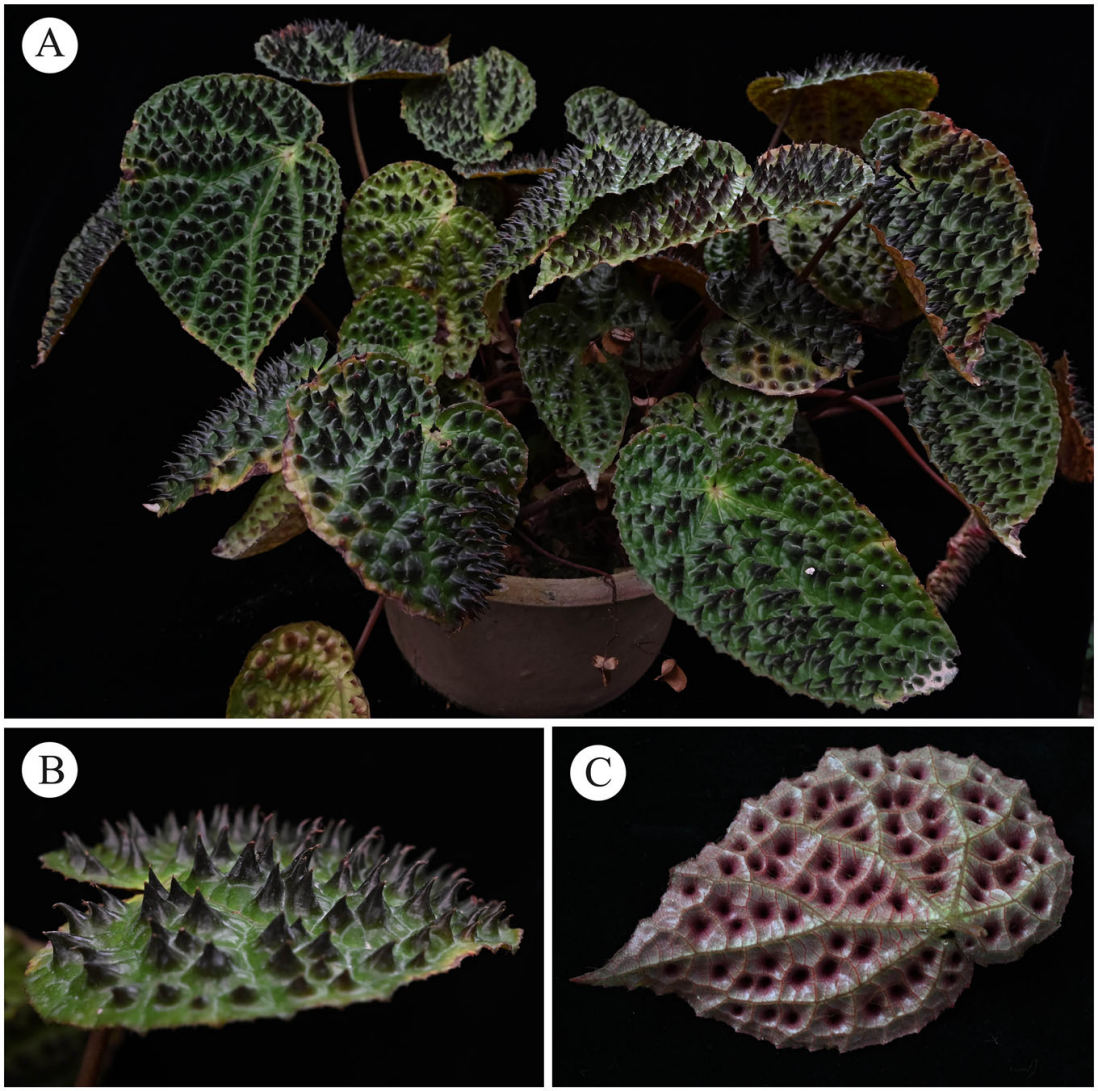
*Begonia ferox* C.-I Peng & Yan Liu. (A) Cultivated plant at fruiting stage; (B) leaf adaxial surface with humps; (C) leaf back surface. All of them were photographed by Zhou in the greenhouse of Guangxi Institute of Botany.

*Begonia* (Begoniaceae) is one of the most diverse plant groups and the fifth or sixth largest angiosperm genus in the world, composed of more than 2000 recognized species names. In the past 20 years, the number of species of the genus has increased dramatically, rising from 80 to 200 species in China (Tian et al. 2018). *Begonia ferox* C.I Peng & Yan Liu (2013) is a perennial herb (Figure 1) of Sect. *Coelocentrum* in the genus *Begonia* (Begoniaceae) (Peng et al. 2013). *Begonia ferox* is mainly distributed on limestone rocks with ample fallen leaves or on exposed rock slopes in evergreen broad-leaved forests in Guangxi Zhuangzu Autonomous Region, Longzhou County only, very rare. The leaf of *B. ferox* covered with rounded swellings like blisters, and intercostal area densely distributed with dark brown and hair-tipped bubbles. Thus, this species has high ornamental value. Due to narrow habitat and commercial collection, it has been facing serious threat. According to the latest red list of Chinese plants, *B. ferox* is assessed as endangered. Therefore, we report the chloroplast genome sequence of *B. ferox* to provide a basis for further study better of this species and deeply exploration of the phylogenetic history of Begoniaceae.

## Materials and methods

The fresh leaves of *B. ferox* were collected from the greenhouse (110°18′17.80″E, 25°04′49.65″N) in Guangxi Institute of Botany, Chinese Academy of Sciences. *B. ferox* sampling was permitted by the Guangxi Science and Technology Base and Special Talents Program, China (Guike AD20325009). The corresponding specimen was deposited at Herbarium of Guangxi Institute of Botany (http://ibk.gxib.cn/, contact person: Yusong Huang, and email: huang-yusong@163.com) under voucher number IBK00445109. Total genomic DNA in leaf tissue samples was extracted using CTAB method following the manufacturers’ protocols (Doyle 1987), was assessed using Agilent 5400 Tapestation (Agilent Technology Ltd., Santa Clara, CA) for quality control (Figure S1). Then, paired-end sequenced by Illumina HiSeq Xten platform (Illumina, San Diego, CA). A total of 18,413,794 clean reads (% reads with ≥ Q30 score = 93.00%) (Figure S2) were generated, then they were assembled by SPAdes 3.11.0 (Bankevich et al. 2012). To check the accuracy of assembly, we used BWA software to compare the Illumina short sequence with the chloroplast sequence, and used SAMtools (Li et al. 2009) depth to calculate the coverage (Figure S3). Finally, the assembled genome was annotated using PGA software (Qu et al. 2019) and further cp genome map was done using OGDRAW (https://chlorobox.mpimp-golm.mpg.de/OGDraw.html) (Greiner et al. 2019), with *Begonia guangxiensis* (GenBank accession no. NC_046385) as the reference genome.

## Results

**Figure 2.**
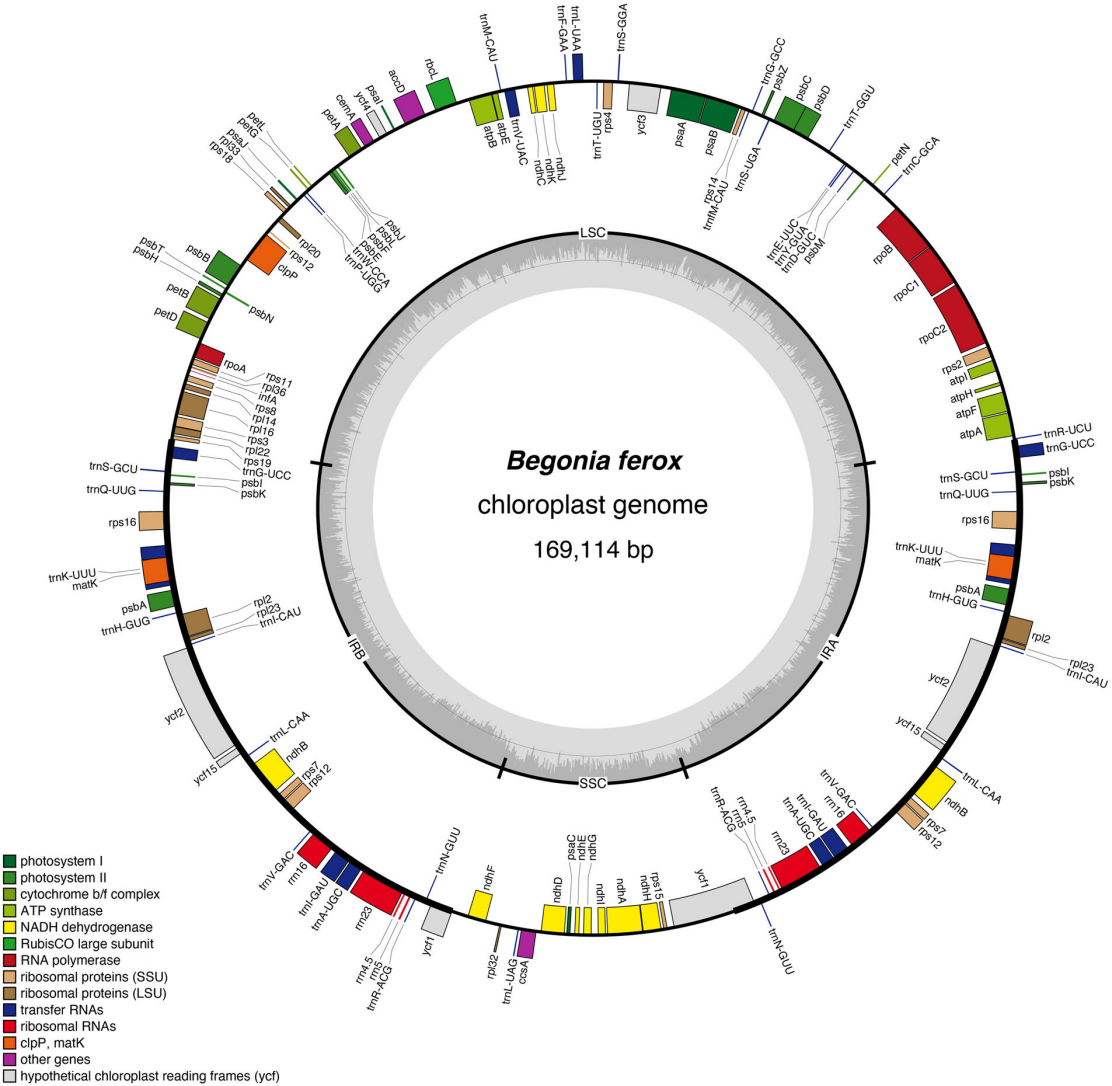
Complete chloroplast (cp) genome map of *B. ferox*. Color coding of genes is based on functional groups they belong to. Dark gray color of inner circle indicates GC content.

**Figure 3.**
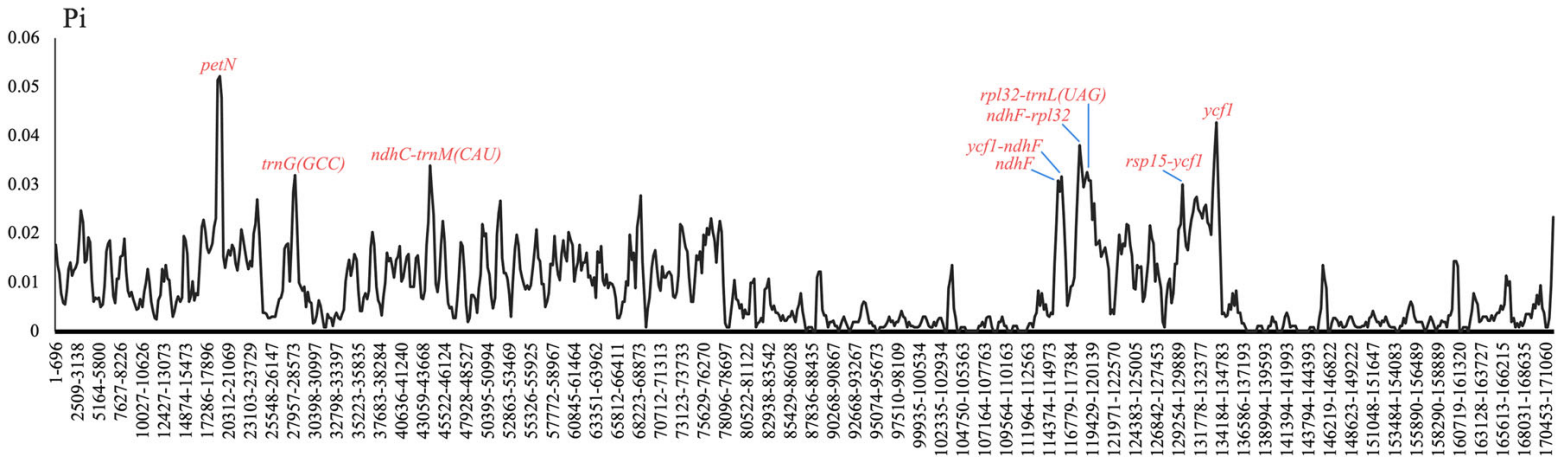
Result of comparative analysis of the nucleotide variability (Pi) values among four *Begonia* species in China.

The chloroplast genome of *B. ferox* (GenBank accession no. NC_067030.1) is an enclosed loop molecule with a length of 169,114 bp (Figure 2). The total GC content is 35.5%. The genome contains a large single-copy (LSC) region of 75,887 bp, a small single-copy (SSC) region of 18,105 bp, and a pair of inverted repeat regions (IRA and IRB) of 37,561 bp. PGA annotation results showed that chloroplast genes contained 144 genes (119 unique genes), including 94 protein-coding genes (80 unique genes), eight rRNAs (four unique genes) and 42 tRNA genes (35 unique genes). Every 28 genes were found to be duplicated in the IR regions, including 12 PCGs genes (*psbI*, *psbK*, *rps16*, *psbA*, *rpl2*, *rpl23*, *ycf2*, *ycf15*, *ndhB*, *rps7*, *rps12*, and *ycf1*), 12 tRNA genes (*trnN-GUU*, *trnR-ACG*, *trnA-UGC*, *trnI-GAU*, *trnV-GAC*, *trnL-CAA*, *trnI-CAU*, *trnH-GUG*, *trnK-UUU*, *trnQ-UUG*, *trnS-GCU*, and *trnG-UCC*), and four rRNA genes (*rRNA4.5*, *rRNA5*, *rRNA16*, and *rRNA23*). The introns were contained in seven protein-coding genes and six tRNA genes, while *ycf3* and *clpP* contain two introns. In particular, a trans-spliced gene *rps12* was detected by CPGview (http://www.1kmpg.cn/cpgview/) (Figure S4). In addition, the cp genomes of four *Begonia* species in China (*B. ferox*, *B. gulongshanensis*, *B. coptidifolia*, and *B. pulchrifolia*) were aligned and compared to screen (used DnaSP) single-nucleotide polymorphisms (SNPs) with higher nucleotide diversity (Ning et al. 2020). As a result, the nucleotide variability (Pi) values were ranged from 0.008 to 0.052 (Figure 3). We found nine of these variable regions (Pi > 0.03) including *petN*, *trnG(GCC*), *ndhC-trnM(CAU)*, *ndhF*, *ycfl-ndhF*, *ndhF-rpl32*, *rpl32-trnL(UAG)*, *rsp15-ycf1*, and *ycf1* showed high level of intrageneric variation.

## Discussion and conclusions

**Figure 4.**
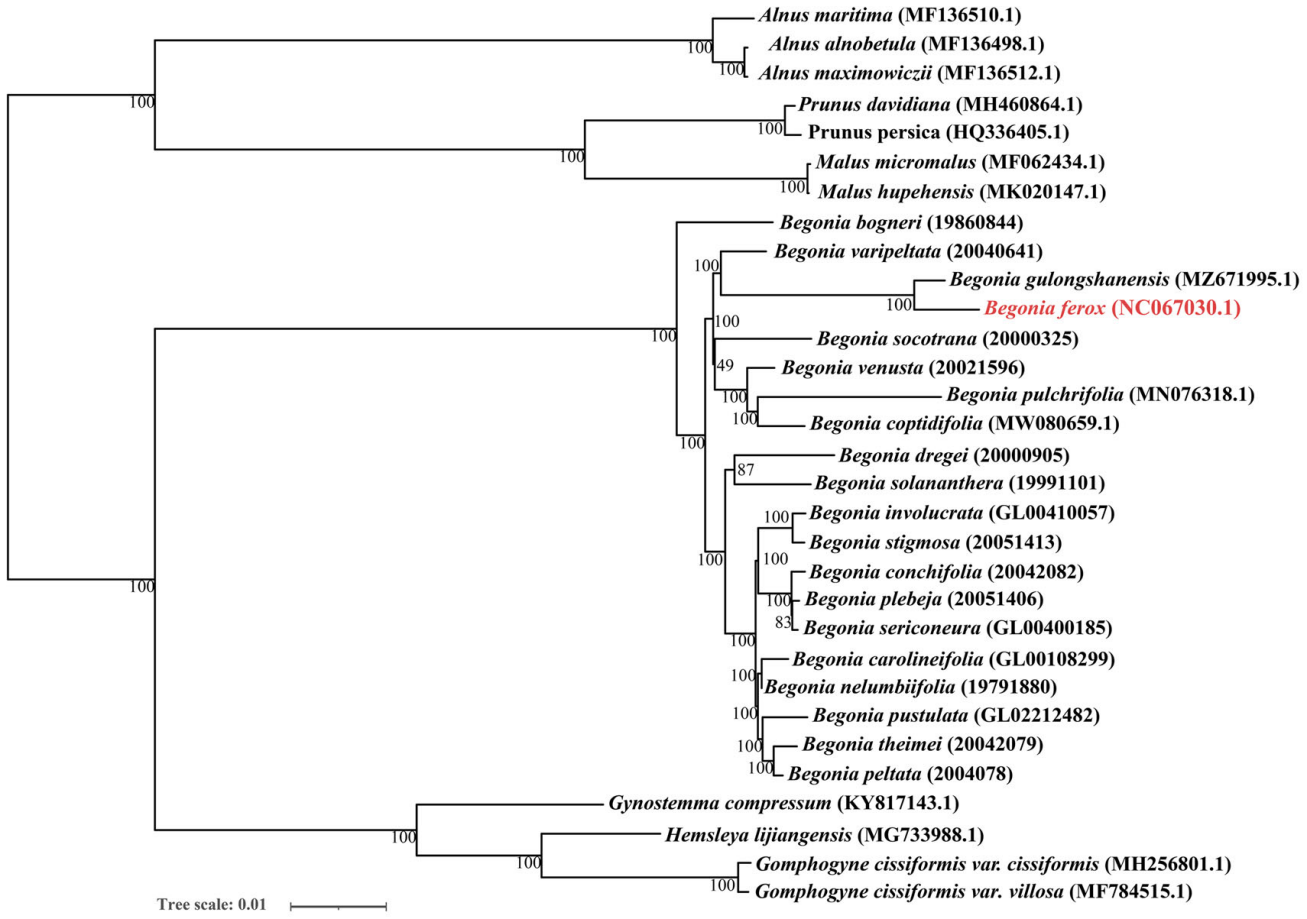
The maximum-likelihood (ML) phylogenetic tree based on the complete chloroplast genomes of *B. ferox* and 30 other published species. Bootstrap support values (1000 repetitions) are shown next to the nodes.

To further analyze the phylogenetic relationship of *B. ferox*, all 31 sequences (14 downloaded from NCBI and 16 downloaded from Dryad (Harrison et al. 2016)) were aligned *via* mafft v7.505 (Katoh and Standley 2013), applying the iterative refinement method (FFT-NS-i) and using default parameter settings (gap opening penalty: 1.53, offset-value: 0.0). Then, the phylogenetic maximum-likelihood (ML) tree was constructed under the GTR + I + G model via W-IQ-TREE (Trifinopoulos et al. 2016) and all nodes were inferred from 1000 bootstrap values, finally visually inspected and manually adjusted in the iTOL (Letunic and Bork 2007). The phylogenetic analysis indicated that *B. ferox* is closely related to *B. gulongshanensis* (Figure 4). We speculated that *B. ferox* and *B. gulongshanensis* both belong to the Sect. *Coelocentrum*, and their type locality is very close, located in the west of Guangxi Zhuang Autonomous Region.

## Supplementary Material

Supplemental Material

## Data Availability

The complete sequence of the chloroplast genome of *B. ferox* has been deposited in GenBank at https://www.ncbi.nlm.nih.gov/, and the accession no. was OP278659. The associated BioProject, SRA, and Bio-Sample numbers are PRJNA872291, SRR21185740, and SAMN30451026, respectively. The data are morally correct and does not violate the protection of human subjects or other valid ethical, privacy, or security concerns.

## References

[CIT0001] Bankevich A, Nurk S, Antipov D, Gurevich AA, Dvorkin M, Kulikov AS, Lesin VM, Nikolenko SI, Pham S, Prjibelski AD, et al. 2012. SPAdes: a new genome assembly algorithm and its applications to single-cell sequencing. J Comput Biol. 19(5):455–477. doi: 10.1089/cmb.2012.0021. 22506599 PMC3342519

[CIT0002] Doyle JJ. 1987. A rapid DNA isolation procedure for small quantities of fresh leaf tissue. Phytochem Bull. 19:11–15.

[CIT0003] Greiner S, Lehwark P, Bock R. 2019. OrganellarGenomeDRAW (OGDRAW) version 1.3.1: expanded toolkit for the graphical visualization of organellar genomes. Nucleic Acids Res. 47(W1):W59–W64. doi: 10.1093/nar/gkz238. 30949694 PMC6602502

[CIT0004] Harrison N, Harrison RJ, Kidner CA. 2016. Comparative analysis of *Begonia* plastid genomes and their utility for species-level phylogenetics. PLOS One. 11(4):e0153248. doi: 10.1371/journal.pone.0153248. 27058864 PMC4825977

[CIT0005] Katoh K, Standley DM. 2013. MAFFT multiple sequence alignment software version 7: improvements in performance and usability. Mol Biol Evol. 30(4):772–780. doi: 10.1093/molbev/mst010. 23329690 PMC3603318

[CIT0006] Letunic I, Bork P. 2007. Interactive Tree Of Life (iTOL): an online tool for phylogenetic tree display and annotation. Bioinformatics. 23(1):127–128. doi: 10.1093/bioinformatics/btl529. 17050570

[CIT0007] Li H, Handsaker B, Wysoker A, Fennell T, Ruan J, Homer N, Marth G, Abecasis G, Durbin R, 1000 Genome Project Data Processing Subgroup. 2009. The Sequence Alignment/Map format and SAMtools. Bioinformatics. 25(16):2078–2079. doi: 10.1093/bioinformatics/btp352. 19505943 PMC2723002

[CIT0008] Ning C, Liao B-S, Cong-Lian L, Shi-Feng LI, Hao Z, Jiang XU, Li X-W, Shi-Lin C. 2020. Complete chloroplast genome of *Salvia plebeia*: organization, specific barcode and phylogenetic analysis. Chin J Nat Med. 18(8):563–572. 32768163 10.1016/S1875-5364(20)30068-6

[CIT0009] Peng CI, Yang HA, Kono Y, Chung KF, Huang YS, Wu WH, Liu Y. 2013. Novelties in *Begonia* sect. *Coelocentrum*: *B. longgangensis* and *B. ferox* from limestone areas in Guangxi, China. Bot Stud. 54(1):44. doi: 10.1186/1999-3110-54-44. 28510884 PMC5430383

[CIT0010] Qu XJ, Moore MJ, Li DZ, Yi TS. 2019. PGA: a software package for rapid, accurate, and flexible batch annotation of plastomes. Plant Methods. 15(1):1–12. doi: 10.1186/s13007-019-0435-7. 31139240 PMC6528300

[CIT0011] Tian D, Xiao Y, Tong Y, Fu N, Liu Q, Li C. 2018. Diversity and conservation of Chinese wild begonias. Plant Divers. 40(3):75–90. doi: 10.1016/j.pld.2018.06.002. 30175289 PMC6114263

[CIT0012] Trifinopoulos J, Nguyen LT, von Haeseler A, Minh BQ. 2016. W-IQ-TREE: a fast online phylogenetic tool for maximum likelihood analysis. Nucleic Acids Res. 44(W1):W232–W235. doi: 10.1093/nar/gkw256. 27084950 PMC4987875

