# Supplementary material for: The complete chloroplast genome sequence of Begonia ferox, an endangered species in China
Source: Mitochondrial DNA B Resour. 2023 Jul 18;8(7):746–50. doi: 10.1080/23802359.2023.2231103 (PMC10355678; doi:10.1080/23802359.2023.2231103)
Supplement: Supplemental Material [file TMDN_A_2231103_SM5753.docx]

**Supplementary materials** for Yangming Zhou, Xuan Yang, Qiongyue Liang, Tao Deng and Xinghua Hu.2023. The complete chloroplast genome sequence of *Begonia ferox*, an endangered species in China.

**
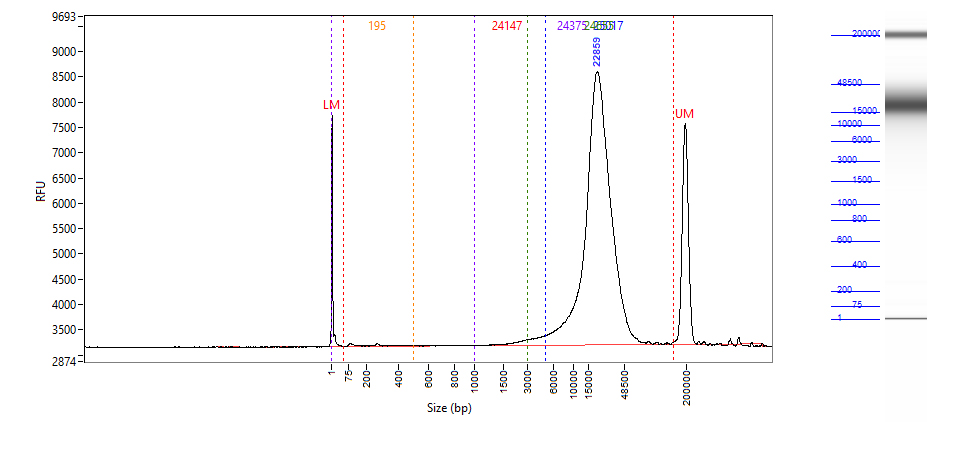
Figure S1. The map of quality control results on an Agilent 5400 Tapestation.** The AATI peak map shows that the DNA sample (*B. ferox*) is qualified. 1. abscissa: fragment distribution of capillary sample where size (bp) reaction is located; 2. The vertical coordinate (RFU) value refers to the real-time fluorescence signal intensity of the sample during capillary separation. The higher the RFU value, the higher the sample concentration; 3. Low Marker/Up Marker: Refers to the reference material (not the fragment of the sample itself) analyzed with the sample, which is used to calibrate the fragment size and concentration of the sample; 4. The right side is the analog glue chart and the corresponding ladder size value.

**Figure S2. The map of sequencing quality distribution.** The map shows that the reads quality score ≥ Q30 score = 93.00%. The sequencing error rate is indicated by "*e*", and the base quality value of the data measured on the Illumina platform is expressed in Q_phred_, then Q_phred_= -10log_10_ (*e*). The abscissa is the base position of reads, and the ordinate is the single base mass value. The first half is the quality distribution of the first end of the double ended sequencing sequence, and the second half is the quality distribution of the other end.


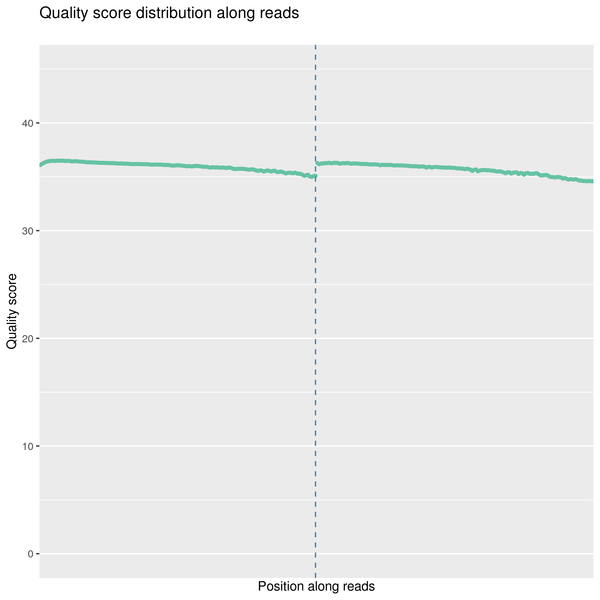


**Figure S3.** Overall coverage depth of the chloroplast genome assembly of *B. ferox*. Used BWA software to compare the illumina short sequence with the chloroplast genome sequence, and finally used SAMtools depth to calculate the coverage. The abscissa is the chloroplast length, and the ordinate is the coverage depth. From the figure, it can be seen that the chloroplast genome sequence has a high coverage of over 100X, data quality is reliable.


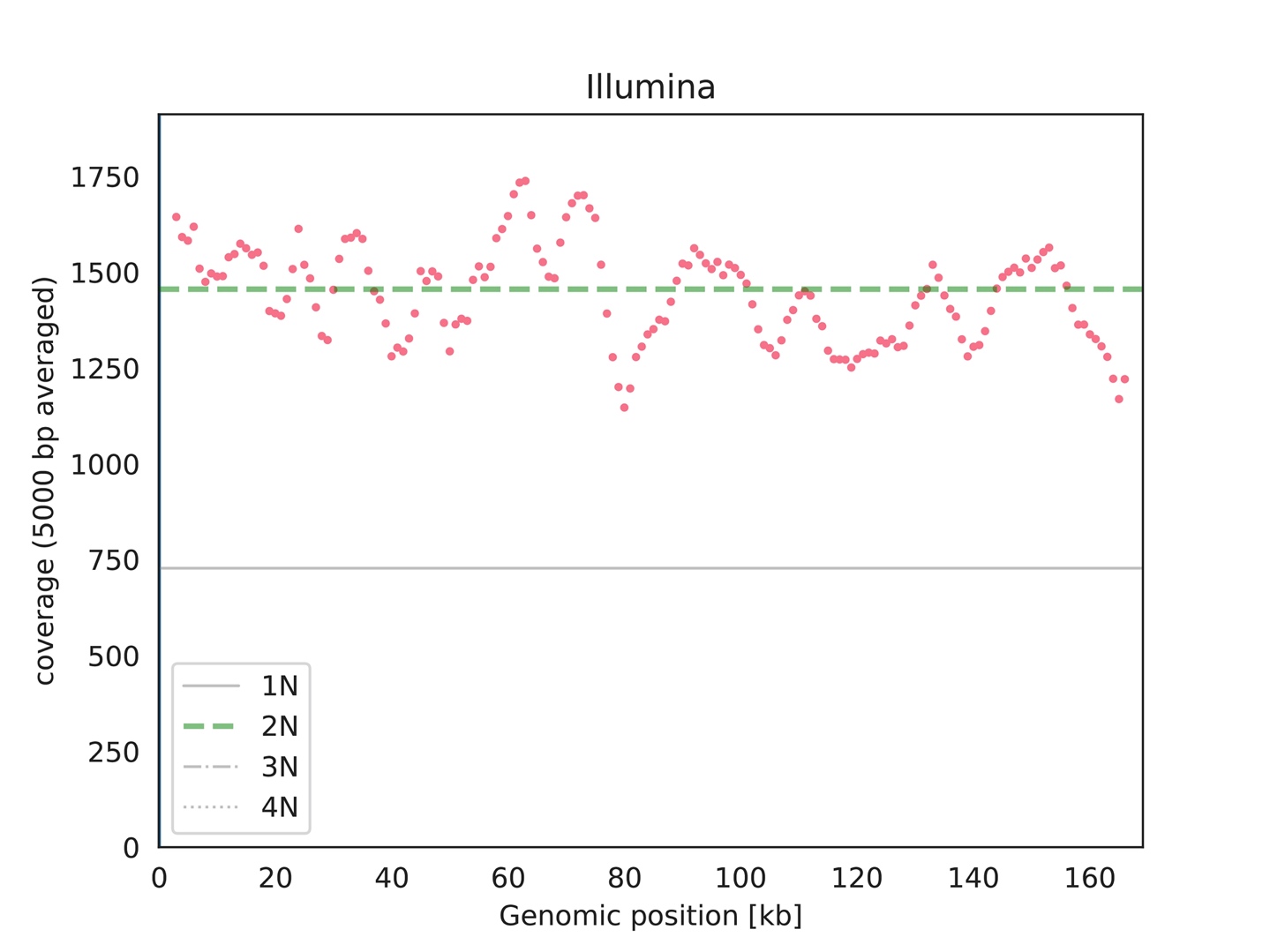


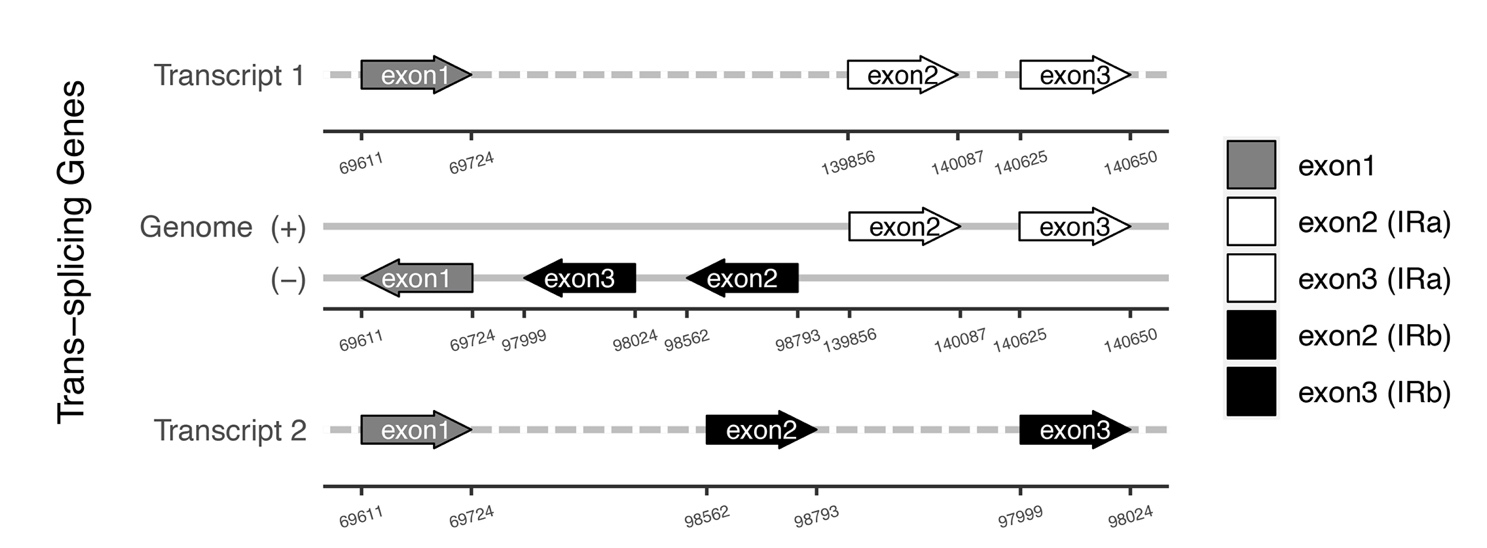
**Figure S4. Schematic map of the trans-splicing gene rps12 in the chloroplast genome of *B. ferox*.** It has three unique exons. Two of them are duplicated as they are located in the IR regions.
